# The functional significance of mitochondrial respiratory chain supercomplexes

**DOI:** 10.15252/embr.202357092

**Published:** 2023-10-12

**Authors:** Andreas Kohler, Antoni Barrientos, Flavia Fontanesi, Martin Ott

**Affiliations:** ^1^ Department of Biochemistry and Biophysics Stockholm University Stockholm Sweden; ^2^ Institute of Molecular Biosciences University of Graz Graz Austria; ^3^ Department of Neurology, Miller School of Medicine University of Miami Miami FL USA; ^4^ Department of Biochemistry and Molecular Biology, Miller School of Medicine University of Miami Miami FL USA; ^5^ Department of Medical Biochemistry and Cell Biology University of Gothenburg Gothenburg Sweden

**Keywords:** bioenergetics, electron transfer, Mitochondria, respiratory chain, supercomplexes, Metabolism, Organelles

## Abstract

The mitochondrial respiratory chain (MRC) is a key energy transducer in eukaryotic cells. Four respiratory chain complexes cooperate in the transfer of electrons derived from various metabolic pathways to molecular oxygen, thereby establishing an electrochemical gradient over the inner mitochondrial membrane that powers ATP synthesis. This electron transport relies on mobile electron carries that functionally connect the complexes. While the individual complexes can operate independently, they are *in situ* organized into large assemblies termed respiratory supercomplexes. Recent structural and functional studies have provided some answers to the question of whether the supercomplex organization confers an advantage for cellular energy conversion. However, the jury is still out, regarding the universality of these claims. In this review, we discuss the current knowledge on the functional significance of MRC supercomplexes, highlight experimental limitations, and suggest potential new strategies to overcome these obstacles.

## Graphical abstract

Mitochondrial respiratory chain complexes can associate into supramolecular structures termed respiratory supercomplexes. This review discusses their structure, assembly and potential physiological functions.

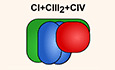

## The mitochondrial respiratory chain

**Figure 1:**
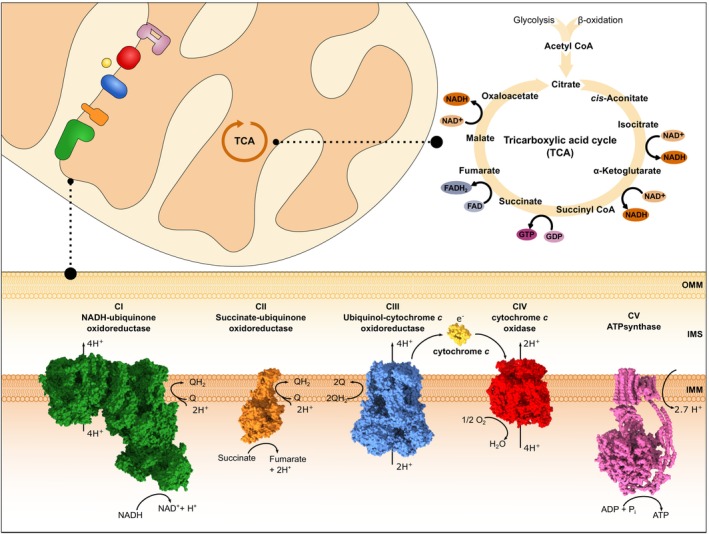
The tricarboxylic acid cycle (TCA) and mitochondrial respiratory chain *Upper Panel*: Acetyl‐CoA is a central metabolite produced via various metabolic reaction pathways. It can subsequently be utilized in the TCA, were among others several molecules of NADH and FADH_2_ are generated. *Lower Panel*: Electrons from these metabolites can be subsequently utilized by the mitochondrial respiratory chain to convert energy in the form of ATP. Structures of the individual respiratory chain complexes and the electron carrier cytochrome *c* are shown, as well as respective redox reactions (PDB CI: 6ZKC; PDB CII: 1ZP0; PDB CIII: 6Q9E; PDB CIV and CytC: 5IY5; PDB CV: 5ARA). IMM, inner mitochondrial membrane; IMS, intermembrane space; OMM, outer mitochondrial membrane.

As the most versatile form of chemical energy in cells, ATP is synthesized in large quantities by mitochondrial activity. Biochemical reactions, many occurring in the cytoplasm, convert carbohydrates and lipids to mitochondrial acetyl‐CoA. This central metabolite is processed in the mitochondrial matrix by the tricarboxylic acid cycle (TCA; Fig 1). Electrons originating from these TCA reactions are passed over to the typically four multiprotein complexes (CI‐CIV) of the electron transport chain. In a process termed oxidative phosphorylation (OXPHOS), the electrons are transported through the mitochondrial respiratory chain (MRC), where they finally reduce molecular oxygen to form water (Fig 1). The energy of this reaction is converted into an electrochemical gradient across the inner mitochondrial membrane (IMM). In a subsequent step, protons flow from the mitochondrial intermembrane space (IMS) back into the matrix, powering the ATP synthase (CV), which condenses ADP and phosphate to form ATP (Fig 1).

NADH feeds two electrons into the NADH dehydrogenase (CI), the first and largest of the MRC multiprotein complexes in mammals, consisting of 45 subunits and comprising a mass of approx. 1 MDa (Kampjut & Sazanov, 2020; Fig 1). Electrons from NADH are thereby transferred to oxidized quinone (Q), a reaction coupled to proton extrusion from the matrix into the IMS via CI's antiporter subunits (Jones *et al*, 2017; Fig 1). Q is an abundant mitochondrial lipid that can be reversibly reduced and oxidized, respectively, and freely diffuses in the IMM. Interestingly, some species, including the model organism *Saccharomyces cerevisiae*, do not contain a conventional CI, but rather possess non‐proton‐translocating peripheral membrane NADH dehydrogenases (Nde1, Nde2, and Ndi1; Joseph‐Horne *et al*, 2001). In addition, electrons extracted from succinate can reduce Q via the succinate dehydrogenase (CII; Fig 1). Whereas CI pumps protons, no H^+^ is translocated by CII. Of note, Q can also be reduced by several other membrane‐bound dehydrogenases (*e.g*., glycerol 3‐phosphate dehydrogenase). The reduced quinone (QH_2_) subsequently transfers two electrons to cytochrome *bc*_1_ reductase (CIII), which exists as an obligate homodimer (CIII_2_; Fig 1) in all species (Stephan & Ott, 2020). CIII couples electron transfer from QH_2_ to cytochrome *c* (Cyt*c*) with the shuttling of H^+^ into the IMS via a mechanism called Q‐cycle (Mitchell, 1975; Osyczka *et al*, 2005). In one such Q‐cycle, two QH_2_ molecules are oxidized to Q, releasing 4 H^+^ into the IMS and reducing two Cyt*c* molecules (Fig 1). Of note, another Q is reduced to QH_2_ in this process, which can subsequently be used for another round of the Q‐cycle. The protein Cyt*c* acts as a mobile electron carrier, with a central, covalently attached heme group that can reversibly bind one electron. Cyt*c* transfers electrons from CIII to Cyt*c* oxidase (CIV), where molecular oxygen is reduced to water (Fig 1). In this last step of the electron transport chain, another 4 H^+^ are pumped into the IMS by oxidizing 4 Cyt*c* molecules (Wikstrom, 1977). Finally, the electrochemical gradient across the IMM formed by the transfer of protons from the mitochondrial matrix into the IMS is used by the ATP synthase (CV), which phosphorylates ADP with inorganic phosphate to ATP, whereby the flow of protons back into the matrix powers ATP synthesis (Mitchell, 1975) by a rotary mechanism of the ATP synthase (Fig 1).

### Connectivity of mitochondrial respiratory chain complexes

Despite detailed insights into the fundamental structural and catalytic properties of the individual MRC complexes, available since last century, their overall structural organization and physiological implications of that organization have been extensively revisited over the last 20 years and are still debated today (Milenkovic *et al*, 2017). Historically, two opposing models for how the complexes are functionally connected were proposed early on, namely, the “solid‐state” and the “liquid‐state” (also referred to as “fluid‐state”) models. The solid‐state model postulates that all components of the MRC (including Q and Cyt*c*) form a single unit, which can catalyze the entire process of mitochondrial respiration. Thereby, Q and Cyt*c* mediate electron transfer via enclosed pathways, never exchanging with the exterior of the unit (Ragan & Heron, 1978). On the contrary, the liquid‐state model proposes that individual complexes reside independently in the IMM. Likewise, Q and Cyt*c* diffuse freely and randomly to transfer electrons between the individual complexes (Hochli & Hackenbrock, 1976; Hackenbrock *et al*, 1986). The liquid‐state model received extensive experimental support, mostly owing to the observation that individual MRC complexes could be purified in active forms and that the small mobile electron carriers are freely diffusing and in large stoichiometric excess over MRC complexes. However, the solid‐state model gained new experimental support. Through the use of mild detergents for mitochondrial membrane solubilization and the establishment of blue native polyacrylamide gel electrophoresis, it was demonstrated that MRC complexes form supramolecular assemblies, termed respiratory chain supercomplexes (SCs) (Cruciat *et al*, 2000; Schägger & Pfeiffer, 2000). SCs were shown to exist in varying stoichiometry in different species, ranging from bacteria to plants, fungi, and animals. Because they exist widely in multiple life forms, they must confer specific, selectable advantages to organisms. In this review, we provide a comprehensive overview of the various forms of mitochondrial SCs, their biogenesis, and their structure and discuss the current understanding of the functional roles of SCs.

## Structures and assembly of different respiratory chain complexes

### Overview of different supercomplex configurations

**Figure 2:**
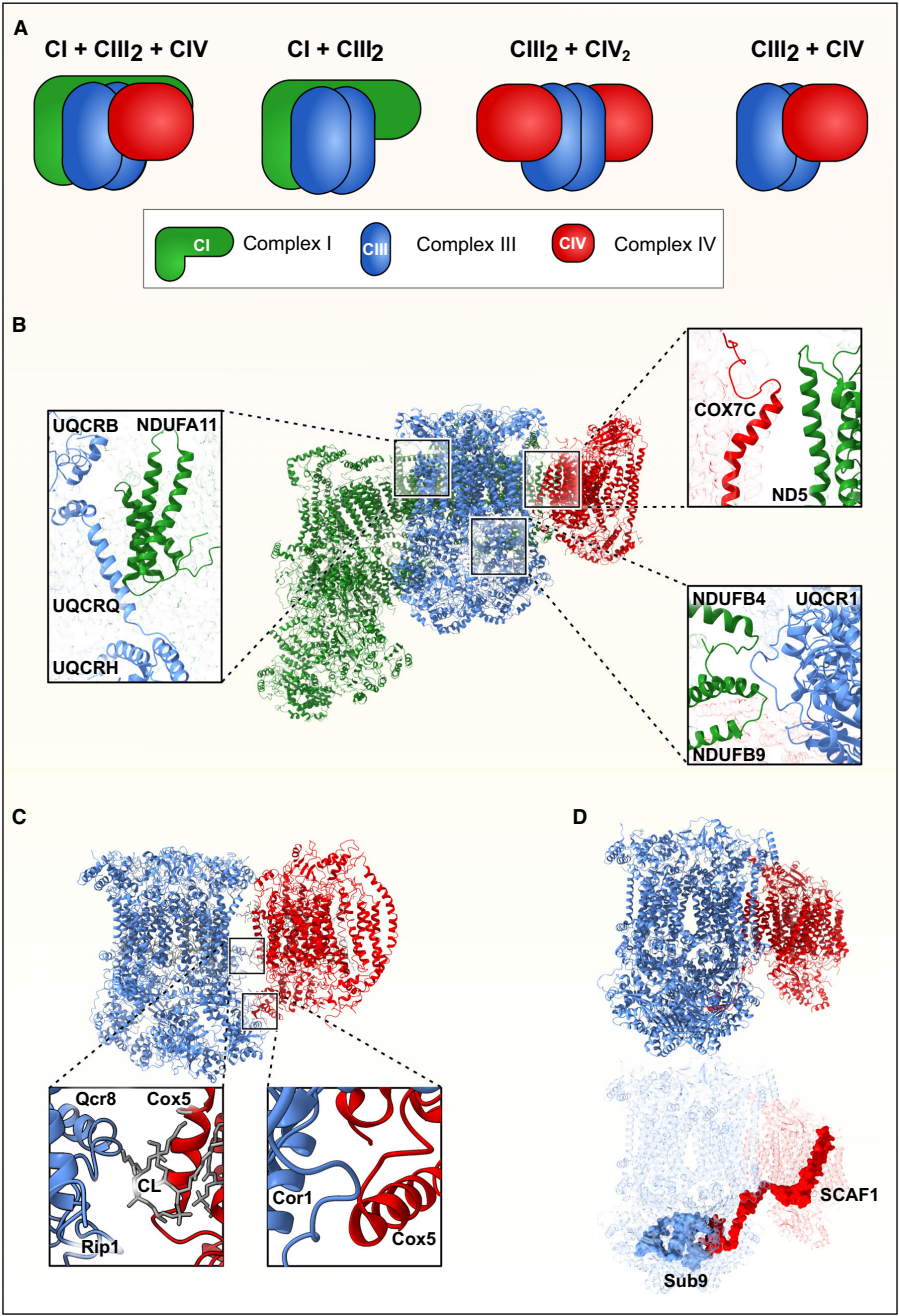
Respiratory chain supercomplex assemblies (A) Stoichiometry of several mitochondrial respiratory chain supercomplexes. (B) Main interaction surfaces of mammalian respirasomes (CI + CIII_2_ + CIV, PDB: 5j4z). Close‐up of interactions between CI (green) and CIII (blue) subunits NDUFA11, UQCRB, UQCRQ, and UQCRH (left panel), as well as NDUFB4 and NDUFB9 with UQCRC1 (lower right panel) are shown, as well as the interaction surface between CIII and CIV (red), formed by the CI subunit ND5 and the CIV subunit COX7C (upper right panel). Visualization is adapted from (Letts *et al*, 2016). (C) Interaction surfaces of the *Saccharomyces cerevisiae* respiratory chain supercomplex CIII_2_ + CIV (PDB: 6YMX). The inner membrane protein–lipid–protein interaction between Rip1 (CIII, blue, cardiolipin (CL) and Cox5 (CIV, red) are shown (left panel), as well as the matrix‐situated protein–protein interaction between Cor1 and Cox5a). Visualization was modified from (Berndtsson *et al*, 2020). (D) Structure of the mammalian respiratory chain supercomplex CIII_2_ + CIV in the unlocked configuration (PDB: 7O3C, upper panel). The lower panel shows a transparent visualization of the same structure to display interactions between the CIII (blue) subunit Sub9 and the CIV (red) subunit SCAF1.

Only a small fraction of CI is present as a free complex in bovine heart mitochondria, and in some studies using cell cultures, the free form of CI is even undetectable (Lobo‐Jarne *et al*, 2018), whereas the rest is found in various SC assemblies with varying stoichiometry (Schägger & Pfeiffer, 2001) (Fig 2A). The most prevalent of these assemblies in human cells is the respirasome, consisting of CI + CIII_2_ + CIV and a size of approx. 1.7 MDa, accounting for approximately half of CI in SCs (Schägger & Pfeiffer, 2001; Gu *et al*, 2016; Letts *et al*, 2016; Wu *et al*, 2016; Lobo‐Jarne & Ugalde, 2018; Fig 2A). Other variants of the respirasome, which have been proposed to contain further copies of CIV (CI + CIII_2_ + CIV_2‐4_), present only minor amounts of CI‐containing SCs. The second most prevalent assembly of CI is the CI + CIII_2_ configuration (Schägger & Pfeiffer, 2001; Fig 2A). Of note, although the stoichiometry of these assemblies is the same between yeasts, plants, and mammals, the structures and frequencies differ (Davies *et al*, 2018). Evidence for the existence of even larger assemblies than the CI + CIII_2_ + CIV_4_ respirasome comes from cryo‐EM studies in HEK293T cells, reporting a symmetrical respiratory megacomplex with the stoichiometry CI_2_ + CIII_2_ + CIV_2_ (Guo *et al*, 2017). Although the authors also speculate that two units of CII could potentially bind to this megacomplex, a corresponding unassigned density could not be observed in their data, and so far, no direct experimental evidence for the participation of CII in supercomplex assembly of metazoa or plants was reported (Caruana & Stroud, 2020). However, a recent study using the ciliate protist *Tetrahymena thermophila* reports a CI + CII + CIII_2_ + CIV_2_ SC, which has important implications in the curvature of the IMM in this organism (Mühleip *et al*, 2023). Of note, this arrangement shows significant differences compared to known mammalian SCs.

Supercomplexes are also formed between CIII and CIV without CI, with the stoichiometries CIII_2_ + CIV_2_ or CIII_2_ + CIV_,_ respectively (Fig 2A). Whereas CIII forms an obligate homodimer (CIII_2_), CIV can occur in its monomeric, as well as in its dimeric (CIV_2_) form.

### The respirasomes

A common feature of respirasomes is the curving of the membrane arm of CI around the CIII dimer, which can already be observed in the isolated CI + CIII_2_ complex (Letts *et al*, 2019). The overall structure of these SCs was similar to the respirasome configuration, but showed at least six open states (inactive form) of CI and one closed state (active form) (Letts *et al*, 2019). In structural studies for both ovine and bovine respirasomes, two different classes had significant variations of the CIV position (Letts *et al*, 2016; Sousa *et al*, 2016). For the ovine respirasome structure, “loose” (active) and “tight” (inactive) conformations were shown, where CIV contacts both CI and CIII in the tight variant but only CI in the loose form (Letts *et al*, 2016). Class 1 of the bovine respirasome is similar to the tight form and represents the most prevalent population and stable form of the respirasome (Gu *et al*, 2016; Letts *et al*, 2016; Letts & Sazanov, 2017). Two main interaction sites are found between CI and CIII_2_, namely, one in the membrane between NDUFA11 and the UQCRB, UQCRQ, and UQCRH subunits of CIII, and a second one in the matrix between NDUFB4, NDUFB9, and CIII subunit UQCRC1 (Fig 2B). Interestingly, the contacts formed between CI and CIII within the respirasome involve so‐called supernumerary subunits. Compared to the 14 conserved CI subunits of bacterial origin, these supernumerary subunits are assumed to be of eukaryotic origin (Gray, 2012). As no CI‐containing supercomplexes have been identified in bacteria so far (however, CIII_2_ + CIV_2_ supercomplexes were described, Kaila & Wikström, 2021), it is feasible that certain CI supernumerary subunits evolved to form respirasomes (Letts & Sazanov, 2017). On the contrary, the contacts between CI and CIV are formed by the evolutionarily conserved core subunit ND5 of CI and the CIV subunit COX7C (Gu *et al*, 2016; Letts *et al*, 2016; Fig 2B).

Different states of the respirasome can be observed when looking at the four major rotational axes of its structure, including the hinge between the matrix and membrane arms of CI, the pivot between CI and CIII_2_, the rotation along the two‐fold‐symmetry axis of CIII_2_, and the internal asymmetric rotation of CIV (Letts *et al*, 2016; Letts & Sazanov, 2017). Whereas the rotation between the matrix and membrane arms of CI may be associated with the regulation of enzymatic activity or may represent different stages in the catalytic cycle (Letts *et al*, 2016; Agip *et al*, 2018; Blaza *et al*, 2018; Kravchuk *et al*, 2022), physiological implications of other conformational states remain unknown. It might be that these different conformations present intermediate states of respirasome assembly or that some configurations (*e.g*., the loose form of the respirasome) are sample preparation artifacts (Letts *et al*, 2016).

The exact mechanism of respirasome formation, particularly involving SC assembly factors, is still a matter of debate. The CIV subunit COX7A has two tissue‐specific isoforms, namely, COX7A1 in the heart and skeletal muscles and COX7A2 in the liver. However, both isoforms can be found in heart‐derived mitochondria, whereas COX7A2 is found in liver mitochondria only. Further, a homolog of COX7A2, COX7A2L, or SCAF1 was described, initially thought to be essential for both respirasome and CIII_2_ + CIV SC formation (Ikeda *et al*, 2013; Lapuente‐Brun *et al*, 2013). It was postulated that SCAF1 is required to form respirasomes in all tissues except in the heart and skeletal muscle (Cogliati *et al*, 2016). However, overwhelming evidence shows that SCAF1 is only required for CIII_2_ + CIV SC formation but not for the respirasome (Davoudi *et al*, 2016; Jha *et al*, 2016; Pérez‐Pérez *et al*, 2016; Sun *et al*, 2016; Williams *et al*, 2016). Although SCAF1 is essential for the assembly of the CIII_2_ + CIV SC (as discussed in more detail below), it does not contribute to the assembly of the respirasome, as the formation of the CIII_2_ + CIV SC is not necessary for respirasome formation (Pérez‐Pérez *et al*, 2016). Likewise, SCAF1 was not detected in the structure of the respirasome. However, a recent analysis of human cells by complexome profiling has led to the identification of two distinct respirasome forms that present slightly different electrophoretic mobility and contain either SCAF1 or COX7A2 isoforms (Fernández‐Vizarra *et al*, 2022).

### Supercomplex CI + CIII_2_ and CIII_2_ + CIV

Although the configuration of CI + CIII_2_ is observed much less frequently in mammalian cells as compared to the respirasome (approx. 17% of total complex I in mitochondria compared to approx. 54% for the respirasome) (Schägger & Pfeiffer, 2001), this arrangement is widely observed across kingdoms (Davies *et al*, 2018).

Recently, high‐resolution structures of *T. thermophila* respiratory chain complexes have been solved, including CI + CIII_2_ (Zhou *et al*, 2022). Interestingly, the overall arrangement is similar to that of mammalian CI + CIII_2_ SCs as discussed above. However, significant differences in the interaction sites were observed, with more contacts in both the matrix and the IMM, as well as an IMS bridge present in *T. thermophila SCs*. Thereby, these interactions cause structural and functional symmetry breaking in CIII_2_. The authors further suggested that no free CI is present in *T. thermophila* and that the CI + CIII_2_ represents the minimal organization of CI (Zhou *et al*, 2022). Moreover, very recent cryo‐EM structures of *Arabidopsis thaliana* CI + CIII_2_ showed a plant‐specific additional interaction site between the complexes compared to ovine CI + CIII_2_ structures, potentially contributing to the unusual stability of the plant SC (Klusch *et al*, 2023). Similar plant‐specific interaction surface between CI and CIII_2_ was also shown for *Vigna radiata* (Maldonado *et al*, 2023).

In the yeast *S. cerevisiae* that lacks a canonical CI, the ratio between the two SC species CIII_2_ + CIV_2_ or CIII_2_ + CIV reflects the cellular energy demands under different growth conditions (Schägger & Pfeiffer, 2000). In particular, the abundance of SC CIII_2_ + CIV_2_ is enhanced in cells propagating in respiratory media and is mainly controlled by the amount of CIV available (Schägger & Pfeiffer, 2000). The first elucidated high‐resolution structure for mitochondrial CIII_2_ + CIV came from *Saccharomyces cerevisiae* (Hartley *et al*, 2019; Rathore *et al*, 2019). The association between CIII_2_ and CIV occurs via protein–protein interactions between the CIII subunit Cor1 and the CIV subunit Cox5, stabilizing the complex from the matrix side, and a protein–lipid–protein interaction between Cor1, cardiolipin, and the CIV subunit Rip1 (Fig 2C). Whereas alanine substitutions in Cor1 abolished the formation of SCs, the reduction of cardiolipin content via deletion of the cardiolipin synthase Crd1 had no direct consequence on SC formation (Pfeiffer *et al*, 2003), showing that the protein–protein interaction between Cor1 and Cox5 is necessary and sufficient for the formation of CIII_2_ + CIV SCs in yeast (Berndtsson *et al*, 2020). However, the yeast and mammalian CIII_2_ + CIV differ substantially in their structure. In yeast, CIV binds to CIII_2_ at the same site as CI does in the respirasome, resulting in different binding modes between CIII and CIV in yeast and mammals (Rathore *et al*, 2019). CIII_2_ + CIV of mammals was observed in two different conformations, a locked and an unlocked state (Vercellino & Sazanov, 2021). In the unlocked state, CIV is peripherally attached to CIII_2,_ and almost 180° rotated compared to the yeast form of this assembly. Whereas the unlocked conformation contained both fully mature CIII_2_ and CIV, the locked form harbors assembly intermediates of CIII_2._

The assembly of mammalian CIII_2_ + CIV has been shown to follow a specific sequence. The assembly factor SCAF1 binds to a premature form of CIII_2_ and drives the docking of CIV to form an intermediate‐locked assembly. This is followed by the maturation of CIII_2_, forming the locked conformation. Subsequently, CIV shifts to the unlocked state, in which the newly added subunits of the mature CIII_2_ form contacts with CIV (Vercellino & Sazanov, 2021; Fig 2D).

The yeast proteins Rcf1 (and its mammalian homolog HIGD2A), as well as Rcf2 (yeast‐specific), were previously suggested to mediate assembly and stability of CIII_2_ + CIV SCs (Chen *et al*, 2012; Strogolova *et al*, 2012; Vukotic *et al*, 2012; Zhou *et al*, 2018). However, Rcf1 was later shown to interact with CIV, thereby regulating the late stages of its assembly (Su *et al*, 2014; Garlich *et al*, 2017). In addition, a recent study showed that Rcf1 and Rcf2 regulate respiration of yeast cells, without significantly affecting the assembly of SCs (Dawitz *et al*, 2020). It was also shown that Rcf1 and Rcf2 are not stoichiometric subunits of CIV, and thus, they were also not present in the cryo‐EM structure of yeast SCs under physiological conditions (Rathore *et al*, 2019). However, Rcf2 could be detected in SC structures under hypoxia‐mimicking conditions (Hartley *et al*, 2020). This is in line with the observation that Rcf1 and Rcf2 regulate CIV activity, in particular under energy‐demanding conditions (Dawitz *et al*, 2020). These results suggest that the primary functions of Rcf1 and Rcf2 are related to the assembly of individual complexes and regulating their function rather than specifically mediating SC formation. In mammals, other relevant HIGD family proteins are HIGD1A and HIGD1C. HIGD1A impacts the assembly of CIII (Timón‐Gómez *et al*, 2020), and in addition, it binds to Cyt*c* and CIV in the respirasome to enhance electron transfer (Hayashi *et al*, 2015; Guerra‐Castellano *et al*, 2018). In agreement, HIGD1A overexpression improves cellular respiration in models of mitochondrial disorders (Nagao *et al*, 2020). Differently, HIGD1C is almost exclusively expressed in glomus cells, where it also interacts with CIV to negatively regulate its activity and facilitate oxygen sensing (Timón‐Gómez *et al*, 2022).

## Physiological functions of supercomplexes

The physiological functions of SCs remain controversial, despite more than two decades of research (Schägger & Pfeiffer, 2000). Several hypotheses regarding their physiological significance were made and will be discussed in this section.

### Free diffusion versus substrate channeling

**Figure 3:**
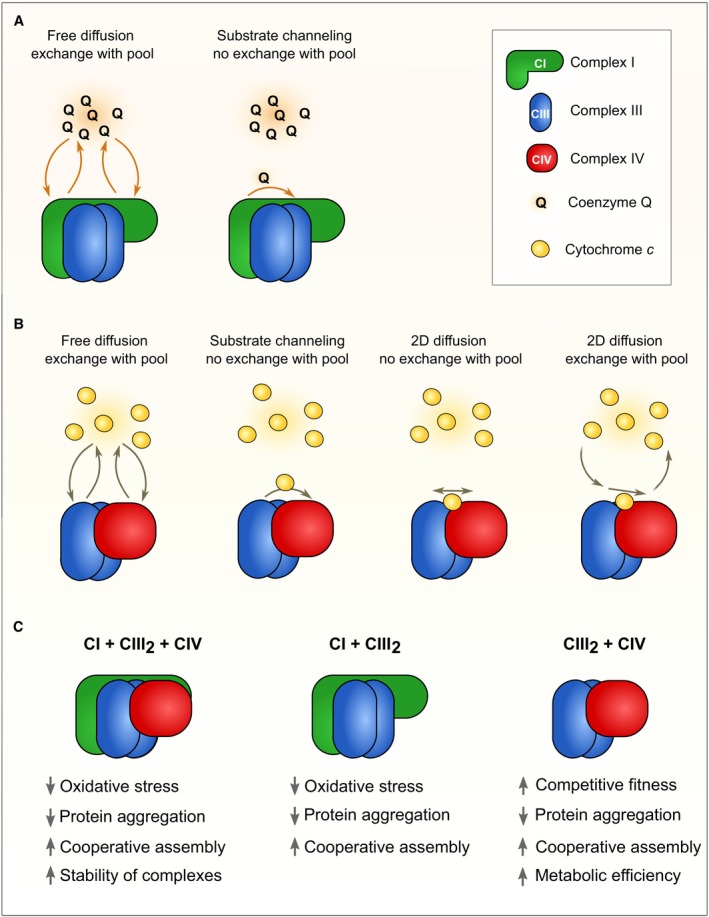
Physiological functions of respiratory chain supercomplexes (A) Comparison between the free diffusion and substrate channeling model for coenzyme Q (Q) within respiratory chain supercomplexes. Arrows indicate the free exchange with the Q pool (free diffusion model, left panel) or the direct Q transfer in the form of substrate channeling (right panel). (B) Comparison of various postulated models for cytochrome *c* shuttling between complex CIII and CIV in respiratory chain supercomplex configuration. Arrows indicate the modality of cytochrome *c* transfer/exchange with the free pool, similar as described for the Q pool above. (C) Potential physiological functions of different respiratory chain supercomplex assemblies. Several of these assigned functions are controversially discussed. Arrows indicate a respective increase or decrease of the indicated physiological functions and pathophysiological effects.

The random collision model suggests that the electron transport within mitochondria is a diffusion‐based process. The mechanistic role of Q to transport electrons from NADH or succinate oxidation to CIII was established in the 1960s, when it was demonstrated that a surprisingly high concentration of Q was found in the IMM, where it behaves as a pool, binding electrons (and protons) to the Q headgroup, and thereby helps to connect electron flow in the MRC (Fig 3A). Because Q is a substantially hydrophobic molecule, owing to the attachment of varying numbers of isoprenyl moieties (Stefely & Pagliarini, 2017), the coenzyme is solved in the lipidic core of the membrane, where it diffuses in the plane of the lipid bilayer. In contrast, Cyt*c* is a protein of roughly 15 kDa molecular weight that carries a covalently attached heme moiety enabling the transport of one electron at a time. Cyt*c* belongs to the class of peripheral membrane proteins. In the absence of transmembrane segments, membrane association stems from its attachment to the IMM by electrostatic interactions with negatively charged phospholipids, particularly cardiolipin, the signature phospholipid of mitochondria (Kinnunen *et al*, 1994). Moreover, biophysical experiments suggested that in addition to electrostatic interactions, also an alternative form of membrane interaction could exist. In this model, an acyl chain of cardiolipin might get inserted into the folded Cyt*c*, giving rise to a tight membrane‐bound form (Rytomaa & Kinnunen, 1995). However, Cyt*c* release from mitochondria during apoptosis is a highly conserved and important event, necessitating that the protein is detached from the IMM (Ott *et al*, 2007). This propensity of Cyt*c,* namely to interact with the IMM via various mechanisms and the well‐documented fact that it can be detached from the membrane to act as a signaling molecule during cell death execution (Garrido *et al*, 2006), complicates the interpretation of how the protein diffuses inside mitochondria. However, the general consent is that Cyt*c* is freely moving in the IMS (Hochman *et al*, 1982), either moving in two dimensions at the level of the IMM, in three dimensions in the IMS, or a mixture of both, whereby the actual ionic strength might dictate the degree of membrane association (Fig 3B).

The discovery of SCs, however, led to the hypothesis that respirasomes might act as solid‐state units, with each respirasome carrying its own Q and Cyt*c* molecules and catalyzing the transfer of electrons from NADH to O_2_ (Fig 3A and B). Flux control analyses take advantage of inhibitors to block a certain step in a reaction pathway. If the effect on the isolated step is equal to the effect on the overall pathway (and hence, the flux control coefficient is 1), this step has complete control over the pathway. Such an analysis has been performed for the NADH oxidation pathway with the inhibitors rotenone and mucidin, and it was shown that CI and CIII behave as a single functioning unit (Blanchi *et al*, 2004). In support of this model, Q and Cyt*c* were found in respirasomes and shown to be able to transfer electrons from NADH to O_2_ (Acín‐Pérez *et al*, 2008). It was therefore proposed that neither Q nor Cyt*c* form homogeneous pools, but instead, each respirasome contains a dedicated small pool of Q and Cyt*c*, which are not exchanged and allow a localized electron transfer via substrate channeling (Lapuente‐Brun *et al*, 2013; Fig 3A and B). Substrate channeling in complexes of enzymes that act sequentially in a pathway is a process where a specific substrate is transferred from one enzymatic activity to the next without allowing free diffusion of the substrate into the bulk solution. For example, the large E1‐E2‐E3 enzymes like pyruvate dehydrogenase or alpha‐ketoglutarate dehydrogenase employ substrate channeling by transferring a covalently bound substrate between the complexes' three enzymatic entities using a highly flexible domain.

Although the substrate channeling hypothesis represents an attractive model for the physiological functions of SCs, experimental evidence against this hypothesis emerged. First, repetitions of flux control experiments showed that the outcome of these experiments strongly depended on the respective inhibitor used (Blaza *et al*, 2014). As certain inhibitors even resulted in a biological meaningless flux control coefficient of > 1, this approach is not a reliable method to analyze physiological functions of SCs (Blaza *et al*, 2014; Milenkovic *et al*, 2017). The authors also measured substrate oxidation using NADH, succinate, or both in submitochondrial particles containing supercomplexes, prepared with and without the addition of Cyt*c* to alter the rate‐limiting step for substrate oxidation. The same system with the same set of supercomplexes showed both additive and non‐additive kinetics (Blaza *et al*, 2014). These results reject the hypothesis of a Q pool partitioning in SCs. Similarly, spectroscopic measurements in living cells also demonstrated that Cyt*c* does not face any barriers to free diffusion (Trouillard *et al*, 2011). In an additional study, an alternative QH_2_ oxidase was inserted into mammalian heart mitochondrial membranes, which then presents a competing pathway for QH_2_ oxidation (Fedor & Hirst, 2018). In the case of substrate channeling, the flux through the competing pathway should be negligible. However, the rate of cyanide‐insensitive NADH:O_2_ oxidoreduction via the alternative QH_2_ oxidase was significantly higher, revealing that CIII/CIV catalysis is rate‐limiting for both, NADH and succinate oxidation, and that Q is not channeled in SCs. In addition, structures of the mammalian respirasomes (Gu *et al*, 2016; Letts *et al*, 2016) and CIII_2_ + CIV SCs (Vercellino & Sazanov, 2021), yeast CIII_2_ + CIV_1‐2_ SCs (Hartley *et al*, 2019; Rathore *et al*, 2019; Berndtsson *et al*, 2020), or plant CIII_2_ + CIV SCs (Maldonado *et al*, 2021) do not contain barriers restricting diffusion of Q or Cyt*c* away from the complexes, features that would be necessary for substrate channeling. Direct tunneling of electrons between CIII_2_ + CIV in the MRC can also be excluded because of a too‐large distance (> 6 nm) between the respective Cyt*c* binding sites (Rathore *et al*, 2019; Vercellino & Sazanov, 2021), allowing electron transfer only by a mobile pool of Cyt*c*. However, bacterial SCs do contain covalently bound Cyt*c*, clearly allowing substrate channeling to transfer electrons from CIII to CIV (Daldal *et al*, 2003; Gong *et al*, 2018; Kao *et al*, 2022).

### Efficiency of electron transfer and competitive fitness

While substrate channeling in a strict definition can be excluded as a possibility to explain the occurrence of SC in mitochondria, it is still feasible that a bioenergetic advantage of SC formation can derive from an increased efficiency of electron transfer between the individual complexes (Fig 3C). One approach to test this hypothesis would be to delete factors essential for the formation of SCs and then determine electron transfer efficiencies. This has been performed for SCAF1, which, as stated above, does not affect the formation of respirasomes, but the assembly of CIII_2_ + CIV SCs. The ablation of SCAF1 did not affect bioenergetics, but rather provoked changes in nutrient sensing (Pérez‐Pérez *et al*, 2016; Lobo‐Jarne & Ugalde, 2018). In contrast, bioenergetic differences were observed in a zebrafish model, where absence of SCAF1 caused abnormal fat deposition and reduction in fertility (García‐Poyatos *et al*, 2020). As further discussed below, controversial results were reported in mouse models, showing either measurable or no effects on energy metabolism upon deletion of SCAF1 (Lapuente‐Brun *et al*, 2013; Mourier *et al*, 2014; Shiba *et al*, 2017).

Moreover, in many systems, including mammalian mitochondria, the respiratory chain complexes assemble in a variety of supercomplexes with varying degrees of stoichiometries, thereby complicating analyses of the bioenergetic efficiency of the different assemblies. Here, yeast offers a unique advantage by lacking CI and having CIII and CIV organized primarily in common supercomplexes. We recently used information from the cryo‐EM structure of *S. cerevisiae* CIII_2_ + CIV_1‐2_ (Rathore *et al*, 2019) to selectively disrupt SCs without affecting individual complexes. We genetically engineered mutants of Cor1, expressed under their endogenous promoter at physiological levels (Berndtsson *et al*, 2020). These point mutations caused the disruption of CIII_2_ + CIV_1‐2_ SCs as the only SC species present in *S. cerevisiae*, without affecting individual complexes. This selective disruption impaired electron transport by impacting the diffusion efficiency of Cyt*c* between the individual complexes (Berndtsson *et al*, 2020). Increased concentrations of Cyt*c* could correct for this defect. Interestingly, this decreased efficiency of electron transfer had no severe consequences on cellular physiology, including unaltered oxidative stress levels, cellular growth, and viability in isolated cultures. However, the disruption of SCs caused massively reduced competitive cellular fitness, which could be restored by the overexpression of Cyt*c*. These results thus demonstrate that the formation of SCs in living aerobic cells increases the efficiency of cellular energy conversion, which promotes an evolutionary advantage that can be selected for (Berndtsson *et al*, 2020) (Fig 3C).

Results on increased electron transfer efficiency established by this work align well with recent mathematical modeling of this process (Stuchebrukhov *et al*, 2020). Interestingly, further work performed with yeast mitochondria in combination with cryo‐EM suggests that this kinetic advantage might be conferred by 2D diffusion of Cyt*c* between CIII_2_ and CIV in SCs rather than 3D diffusion between separated, individual complexes (Moe *et al*, 2021) (Fig 3B). In line with a previous proposal (Pérez‐Mejías *et al*, 2019), it was suggested that CIII_2_ + CIV SC formation provides a negatively charged surface patch on top of this SC, on which the positively charged Cyt*c* can “roll”. These structural properties can also be found in the mammalian version of this complex (Vercellino & Sazanov, 2021). Hence, this mechanisms might present an alternative to substrate channeling, for which diffusion of Cyt*c* within the SC is favored by electrostatic interactions, but still allows a free exchange with the universal soluble Cyt*c* pool (Moe *et al*, 2021; Vercellino & Sazanov, 2021, 2022; Fig 3B). However, the significance of an electrostatic interaction for 2D diffusion restricted to SCs depends on the ionic strength of the local environment and the lipid composition of the IMM. Currently, these aspects have not been firmly determined, but are essential to establish the relevance of such a 2D diffusion. Moreover, whether physiological or environmental factors could modulate the ratio of Cyt*c* 2D versus 3D diffusion has not been investigated. In addition, limiting the 3D diffusion of Cyt*c* impairs mitochondrial function and cellular viability (Toth *et al*, 2020). Of note, these results were obtained by reducing the mobility of the whole Cyt*c* pool and not only a potential subpopulation interacting with SCs. In sum, current studies demonstrate that the formation of CIII_2_ + CIV SCs increases the efficiency of electron transport via Cyt*c*. However, the exact modalities and biophysical properties establishing this advantage remain to be investigated.

### Mitochondrial respiratory complex stability and assembly

The structural interdependence between MRC complexes was first observed in cells from patients with mitochondrial diseases, where mutations affecting CIII or CIV frequently lead to a combined CI deficiency (Lamantea *et al*, 2002; Fernandez‐Vizarra *et al*, 2007), suggesting that SCs may play roles in CI stability. The observation that, in a CIII mutant, CI levels could be restored by treatment with antioxidants, while SC‐associated CI is not affected by high ROS levels (Diaz *et al*, 2012), suggests that only free CI is highly sensitive to ROS‐induced damage and degradation.

Another hypothesis on the physiological significance of SCs suggested that these large assemblies are scaffolds for the late stages of CI biogenesis involving the incorporation of the NADH module to the CI peripheral arm (Moreno‐Lastres *et al*, 2012). However, this hypothesis has been challenged by kinetic studies of CI assembly, where a small amount of fully assembled CI could be detected before the formation of SCs (Guerrero‐Castillo *et al*, 2017). More recently, it was shown that the lack of CIII prevents the incorporation of CI NADH module from completing the functional enzyme (Protasoni *et al*, 2020). Moreover, the association of the late assembly CI precursor with CIII and/or CIV was detected in mutant cell lines and animal models (Alston *et al*, 2018; Adjobo‐Hermans *et al*, 2020; Fang *et al*, 2021). Additionally, data supporting the interaction of CIII and CIV subunits and assembly modules directly in the SCs have been reported (Lazarou *et al*, 2009; Lobo‐Jarne *et al*, 2020; Timón‐Gómez *et al*, 2020). Taken together, increasing evidence supports the existence of distinct but interconnected assembly pathways for free complexes and SCs, a model referred to as the cooperative assembly model (Fernández‐Vizarra & Ugalde, 2022).

### Mitochondrial respiratory chain organization and metabolic adaptation

The plasticity model of mammalian MRC organization proposes that individual complexes assemble into SCs in a dynamic manner to adapt to changing cellular metabolic needs (Acín‐Pérez *et al*, 2008). However, despite the variety of MRC complexes distribution in free form and SCs observed in different cell types and tissues (Lapuente‐Brun *et al*, 2013), the data reflect steady‐state levels and the dynamic nature of the MRC organization has not been experimentally demonstrated. Alternatively, adaptation to the cellular energetic requirements could be provided by the existence of interconnected assembly pathways, where incorporation of late assembly catalytic modules into individual complex or SC pre‐assembled intermediates could modulate MRC organization (Fernández‐Vizarra & Ugalde, 2022). Although the mechanisms behind these models are yet to be fully disclosed, both scenarios suggest the involvement of SC assembly/disassembly factors acting as sensors or assembly checkpoints. As mentioned above, the only dedicated SC assembly factor identified to date is SCAF1 (Cogliati *et al*, 2016). There is a general agreement that in all cell lines and model organisms, the absence of or mutations in *SCAF1* lead to the lack of SC CIII_2_ + CIV formation, while allowing the assembly of respirasomes (Cogliati *et al*, 2016; Pérez‐Pérez *et al*, 2016; Lobo‐Jarne & Ugalde, 2018). It is also established that the stability of respirasomes and, particularly, larger SC structures is compromised to different extents depending on the cell type and tissue (Cogliati *et al*, 2016; Pérez‐Pérez *et al*, 2016; Williams *et al*, 2016; Lobo‐Jarne & Ugalde, 2018). On the contrary, the question of whether the changes in SC organization promoted by the absence of SCAF1 ultimately lead to a metabolic adaptation remains to be fully answered. Some studies concluded that SCAF1‐dependent MRC remodeling is not essential, neither to maintain mitochondrial bioenergetics nor to cope with acute cellular stresses (hypoxia or oxidative stress) in HEK293T cells (Pérez‐Pérez *et al*, 2016; Lobo‐Jarne & Ugalde, 2018). Other studies showed that in U2OS cells, ER stress and glucose deprivation stimulate mitochondrial bioenergetics and SC formation through protein kinase R‐like ER kinase (PERK) by enhancing SCAF1 levels through the PERK‐eIF2α‐ATF4 axis (Balsa *et al*, 2019). Likewise, increased SC levels were associated with mitochondrial efficiency and growth in severely hypoxic pancreatic cancer (Hollinshead *et al*, 2020). An inconsistent picture was also obtained from the study of animal models. These studies have shown that SCAF1 promotes SC formation as well as metabolic efficiency in zebrafish (García‐Poyatos *et al*, 2020) and in some mouse models (Lapuente‐Brun *et al*, 2013; Shiba *et al*, 2017) but not others (Mourier *et al*, 2014). One of these studies showed that *SCAF1* KO mice exhibit lower blood glucose levels after insulin or pyruvate injection, which suggested that the protein could be involved in the regulation of glucose homeostasis (Shiba *et al*, 2017). However, whether the physiological manifestations correlated with or were the cause of altered SC formation was not fully defined. Work in zebrafish models showed that SCAF1‐deficient animals were smaller in size and had abnormal fat deposition, phenotypes that were rescued by doubling the food supply (García‐Poyatos *et al*, 2020). This phenotype rescue correlated with improved bioenergetics; however, possible SC‐independent roles of SCAF1 in fat metabolism in fish were not fully discarded.

Two more recent studies have added support to the concept of MRC organization‐dependent metabolic remodeling. One study showed that two separate MRC organizations co‐exist in human cells and post‐mitotic tissues, which the authors called C‐MRC and S‐MRC. These are defined by the preferential expression of the CIV COX7A isoforms, COX7A2 and SCAF1 (Fernández‐Vizarra *et al*, 2022), respectively. The SCAF1‐dependent S‐MRC organization is characterized by the presence of the SCAF1‐containing respirasome, which accounts for approximately 50% of total CIII and CIV levels. At the same time, the remaining CIII and CIV are equally distributed between the CIII_2_ + CIV SC and free complexes. On the contrary, the COX7A2‐dependent C‐MRC organization displays a relatively low amount of the COX7A2‐containing respirasome, no CIII_2_ + CIV SC, and abundant free CIII (~ 60% of total CIII) and CIV (~ 80% of total CIV). The exclusive presence of one configuration or the other in knock‐out cells of the corresponding isoform led to some changes in mitochondrial bioenergetics. However, no differences in respiratory parameters were observed between *SCAF1* or *COX7A2* knock‐out and wild‐type cells, where the two MRC organizations co‐exist. The increment in respiratory rate supported by the addition of succinate (a CII substrate) to permeabilized cells already respiring CI substrates was shown to be lower in cells expressing the S‐MRC compared to C‐MRC, suggesting that the electrons from CI saturate the downstream MRC segment (Q to CIV) at a greater extent in the S configuration. Preferential utilization of the NADH versus FADH_2_ route parallels with the fraction of total CIII and CIV distributed in respirasomes. This suggests the existence of a kinetic advantage for electron transfer within SCs possibly linked to the proximity of electron carrier binding sites. However, NADH‐ and FADH_2_‐linked respiration are not fully additive in either configuration, arguing against Q pool partitioning. Moreover, the lack of COX7A2 causes a decrease in total CIV amount and enzymatic activity by 50%, which, although it does not affect endogenous and coupled respiration, reduces spare respiratory capacity. This relevant confounding variable can contribute to the observed decrease in CII minus CI‐ and ATP‐driven respiration in S‐MRC cells. Lastly, the authors showed that stimulation of pyruvate dehydrogenase (PDH) activity to promote a switch toward oxidative metabolism in human fibroblasts reduces SCAF1 steady‐state levels and favors the C‐MRC configuration. This observation establishes a direct link between the MRC organization and cellular metabolism, controlled by PDH activity. Intriguingly, while the study proposes that a metabolic shift toward oxidative metabolism destabilizes SCAF1, data from human and mouse tissues indicate that SCAF1 expression is induced by PGC1α and enhances respiratory capacity (Benegiamo *et al*, 2022).

A study in humans has shown significant genetic variation in SCAF1 in several tissues, most prominently in skeletal muscle (Benegiamo *et al*, 2022). The most significant variant was an insertion in the 3′ untranslated region of the gene that creates a short‐repeated sequence and enhances SCAF1 expression. Human myotubes carrying this genetic variant have enhanced SC levels and respiratory capacity. One would expect that the S‐MRC would accumulate in these myotubes, but this was not determined. Nevertheless, an important finding was that this variant is associated with lower body fat and improved cardiorespiratory fitness in humans (Benegiamo *et al*, 2022). This observation was reproduced in a mouse model, where it was also shown that SCAF1 and SCAF1‐containing SCs are induced upon exercise, specifically in the skeletal muscle, in a manner independent of PGC1α (Benegiamo *et al*, 2022). Taken together, these studies support the conclusion that SCAF1 and SC levels correlate with metabolic fitness (Fig 3C), although the cellular context may modulate the nuances of this correlation.

### Other proposed functions of respiratory chain supercomplexes

Most studies on SCs mention that the limitation of oxidative stress could be a function of these assemblies. However, only very limited evidence for such a role was provided. CI and CIII are the main production sites of reactive oxygen species in mitochondria (Murphy, 2009), and hence, it was postulated that the formation of the respirasome somehow alleviates oxidative stress from these sites. Indeed, an *in vitro* study suggests that the dissociation of CI + CIII_2_ SCs increases oxidative stress (Maranzana *et al*, 2013). In line with these results, it was observed that the higher abundance of free CI in astrocytes compared to neurons correlates with higher oxidative stress found in astrocytes (Lopez‐Fabuel *et al*, 2016). Unfortunately, a molecular mechanism for such a potential reduction of reactive oxygen species by SC formation has not been demonstrated, and hence, this hypothesis is only based on correlations. A causative role of SC formation to limit oxidative stress remains to be determined.

Finally, it was also proposed that SCs prevent the aggregation of proteins in the IMM (Blaza *et al*, 2014). Although this is feasible, as the IMM presents one of the most protein‐dense regions of a cell, direct experimental evidence for this hypothesis is missing.

## Summary

Since the discovery of SCs more than 20 years ago (Schägger & Pfeiffer, 2000), significant advances in our understanding of these gigantic assemblies have been made. In particular, the latest work on the structural characterization of SCs presented a giant leap in the field (Gu *et al*, 2016; Letts *et al*, 2016; Hartley *et al*, 2019; Rathore *et al*, 2019; Vercellino & Sazanov, 2021; Zhou *et al*, 2022; Klusch *et al*, 2023; Maldonado *et al*, 2023). Still, the physiological functions of SCs remain a matter of debate (Milenkovic *et al*, 2017). Recent reports have indicated that SC formation primarily enhances the efficiency of electron transport. The emerging picture favors a model in which SC formation, as such, does not modify the maximal activity of individual complexes (Mourier *et al*, 2014; Berndtsson *et al*, 2020), but optimizes their cooperation in the context of the overall pathway.

Supercomplex formation constitutes another example for a growing theme of gathering functionally related activities in common complexes or into proximal localization. This strategy, which is widely employed in life, is used extensively in mitochondria (Linden *et al*, 2020; Schulte *et al*, 2023) as exemplified by a supercomplex collecting enzymes mediating Q biosynthesis (Marbois *et al*, 2009), the establishment of membrane subdomains where early steps of MRC biogenesis are organized (Singh *et al*, 2020) and mitochondrial RNA granules (Antonicka *et al*, 2013; Jourdain *et al*, 2013), executing early steps of mtRNA maturation. However, while all these organizations might not be essential for these pathways to occur, they likely improve the overall biochemical efficiency, possibly only with a small, but nevertheless selectable, margin. While these small beneficial margins may be neglectable or even sometimes undetectable in experimental setups and under laboratory conditions, they have impacted selection during evolution and seem to affect performance in mammalian tissues. Particularly in the context of evolutionary pressure via competition in natural environments and in stress scenarios (*e.g*., high temperature, hypoxic conditions, limited nutrients), these subtle bioenergetic advantages of SC formation can be the basis for selection. Employing the recent structural insights into respiratory SCs should allow engineering of further sets of genetic mutants to fully unravel the intricacies and physiological impact of SC formation.

**In need of answers**

- To fully unravel the functional interplay of respiratory chain complexes in SCs and its mechanistic benefit, it might be necessary to reconstitute purified SCs into proteoliposomes to study their performance in a minimal system, free from confounding secondary variables.
- The functional connectivity of the respiratory chain by mobile electron carriers is likely a key aspect of SC/respirasome formation. It would be important to determine the absolute and relative concentrations of Cyt*c* or Q, and RCs in order to test how they might determine the dependency on SC/respirasome formation to achieve optimal electron transport rates.
- While this manuscript was under editorial proof, an article was published where a more than 50% reduction in SC and respirasome formation was achieved in a mouse model, without overly perturbing respiration or physiology (Milenkovic *et al*, 2023). Hence, it would be desirable for future studies to generate mutants in higher organisms, where the formation of SCs and respirasomes is completely abolished.
- Once these mutants are available, it would be very interesting to score how they impact stress tolerance and competitive fitness.
- The interdependency of assembly of the different respiratory chain complexes has suggested that mature complexes might facilitate assembly of the partner complex through direct contacts. However, assembly also occurs in the absence of the partner complex. To unravel the contribution for the co‐assembly pathways for overall assembly, efficiency will be an important aspect of future research.

## Author contributions

**Andreas Kohler:** Conceptualization; writing – original draft; writing – review and editing. **Antoni Barrientos:** Conceptualization; writing – original draft; writing – review and editing. **Flavia Fontanesi:** Conceptualization; writing – original draft; writing – review and editing. **Martin Ott:** Conceptualization; writing – original draft; writing – review and editing.

## Disclosure and competing interests statement

The authors declare that they have no conflict of interest.
